# Endovascular Treatment of Ruptured Renal Artery Aneurysm: A Case-Based Literature Review

**DOI:** 10.1155/2019/3738910

**Published:** 2019-02-06

**Authors:** Yub Raj Sedhai, Soney Basnyat, Tawseef Dar, Deepak Acharya

**Affiliations:** ^1^Clinical Assistant Professor of Medicine, Virginia Commonwealth University, School of Medicine, Richmond, VA, USA; ^2^Resident, Internal Medicine, St. Mary Mercy Hospital, Livonia, MI, USA; ^3^Nuclear Cardiology Clinical and Research Fellow, Massachusetts General Hospital Corrigan Minehan Heart Center, Harvard Medical School, Boston, MA, USA; ^4^Resident, Internal Medicine, Interfaith Medical Center, Brooklyn, NY, USA

## Abstract

Renal artery aneurysms are extremely uncommon with a reported incidence of less than one percent in general population. They are being more frequently detected due to increasing availability and use of abdominal imaging. Renal artery aneurysm rupture is an extremely unusual cause of acute flank pain with hemodynamic instability. Given the rarity of diagnoses, clinicians may not consider and address this ruptured renal artery aneurysm early which can potentially lead to adverse clinical outcomes. We report the case of a 55-year-old male who presented with retroperitoneal bleeding from a ruptured aneurysm of the interlobular branch of renal artery. He was endovascularly treated with coil embolization. We have also reviewed the inherent literature.

## 1. Case Presentation

A 55-year-old male with the history of end-stage renal disease (ESRD) on triweekly hemodialysis presented with right flank pain that began in the morning on the day of presentation. Pain was sudden in onset, cramping in type, continuous with intermittent exacerbation, and radiated to the groin. The patient denied history of fever, nausea, vomiting, and pain elsewhere in the back and the abdomen and denied prior history of similar pain in the back or abdomen. He denied diarrhea or constipation. Change in the color of urine could not be assessed as he had been anuric for two years. He had history of diabetes, hypertension, hyperlipidemia, and end-stage renal disease secondary to diabetic nephropathy and hypertensive nephrosclerosis. He did not have history of abdominal trauma, surgery, or renal biopsy. Clinical assessment one day back during last dialysis was normal, and the dialysis session was uneventful. Evaluation of vitals in the emergency department revealed sinus tachycardia with heart rate 120 bpm, blood pressure was 130/70 mmHg, and respiratory rate was 16 breaths per minute with saturation of 95% on room air. Jugular venous pressure was not raised. Examination of the abdomen including back revealed tenderness of right renal angle and flank. Abdominal examination was otherwise unremarkable. Systemic examination of cardiovascular, respiratory, and neurologic systems was within normal limits.

## 2. Investigations

**Figure 1:**
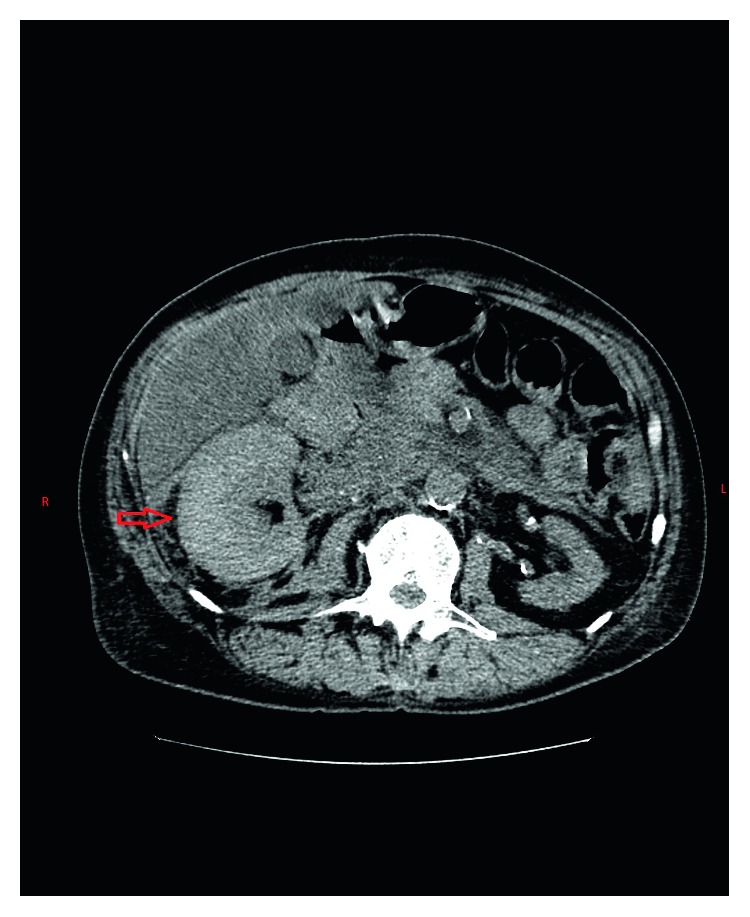
Axial section of noncontrast CT scan of the abdomen showing an enlarged right kidney (marked in red arrow) suggesting a potential collection within or outside the renal capsule.

**Figure 2:**
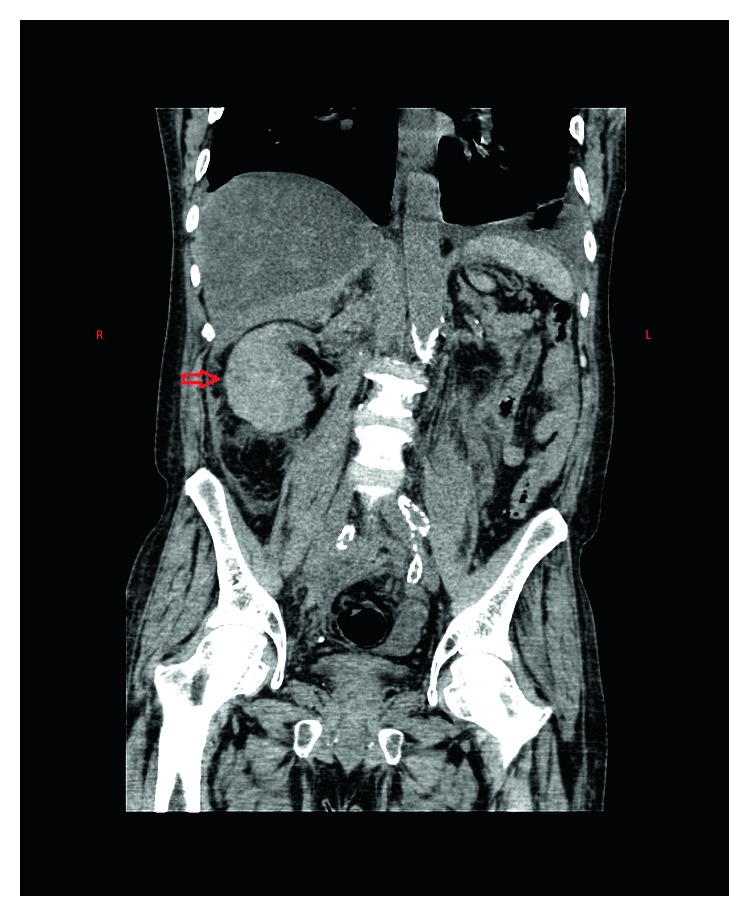
Coronal section of the noncontrast CT scan of the abdomen showing an enlarged right kidney (marked in red arrow) suggesting a potential collection with or outside the renal capsule.

Laboratory workup revealed hemoglobin of 6.5 grams/deciliter with hematocrit of 28 percent. His baseline hemoglobin and hematocrit were 10.5 grams/dl and 38 percent, respectively, in a recent hemogram performed one month back. A wide range of differential diagnoses was considered at the initial presentation. It included urinary calculus, acute pyelonephritis, renal infarction, and musculoskeletal pain. However, given the acute drop in hematocrit with severe pain and sinus tachycardia in face of multiple vascular risk factors also raised the remote yet alarming possibility of abdominal aortic aneurysm rupture. Point of care ultrasound performed at bedside revealed a heterogenous echogenicity around the right renal parenchyma. An emergent computed tomography (CT) of the abdomen was performed. It revealed large retroperitoneal hemorrhage surrounding the right kidney and expanding into the pelvis through the retroperitoneal space (Figures 1 and 2). The source of bleeding including abdominal aortic aneurysm could not be identified. The patient was initially treated conservatively anticipating that bleeding will cease from tamponade effect in the retroperitoneal space. He received four units of packed red blood cells transfusion for the next two days without an appropriate hemoglobin response. The patient started developing features consistent with hemorrhagic shock. A repeat CT scan of the abdomen on the second day of admission revealed expanding hematoma within and outside the renal capsule suggesting a renal vs renovascular source of bleeding. At this point, a decision was made to perform abdominal aortography with selective renal angiography.

## 3. Treatment/Intervention

**Figure 3:**
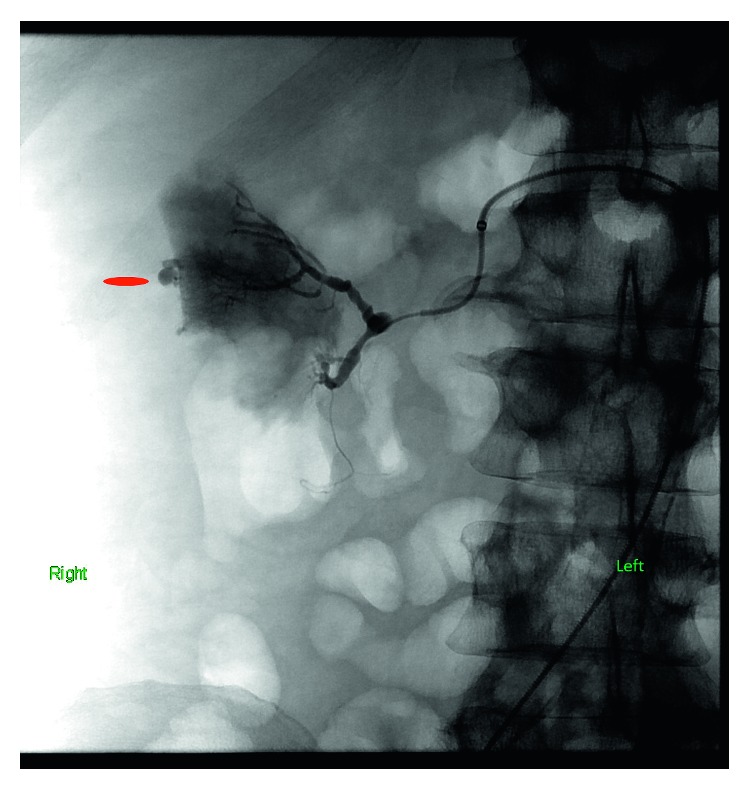
Selective right renal angiogram showing renal artery branch aneurysm with extravascular leakage of contrast.

**Figure 4:**
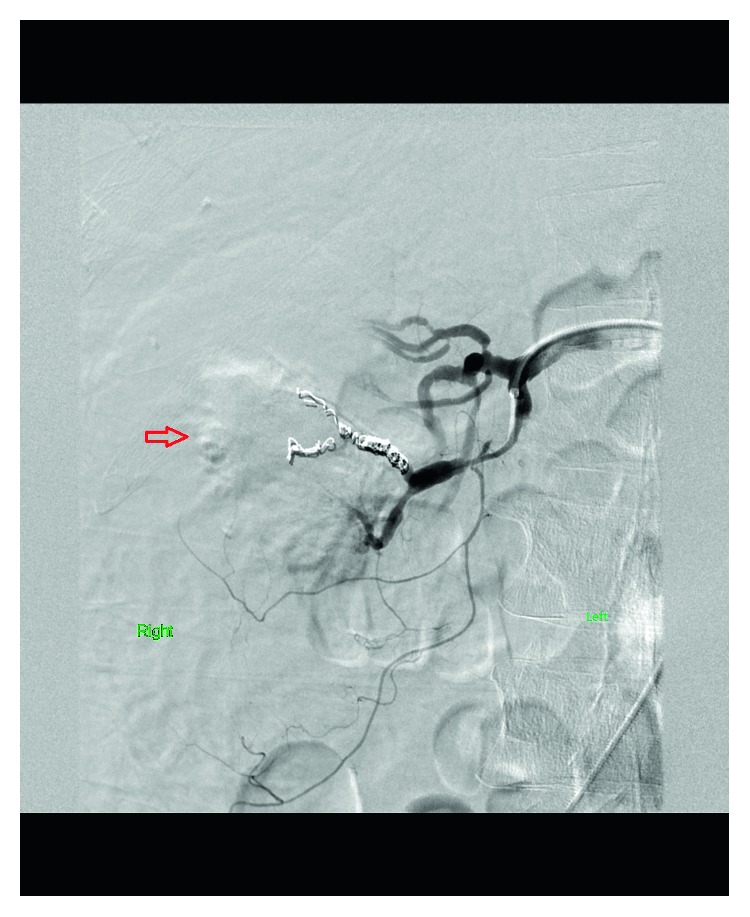
Coil embolization of the feeding interlobar branches and the posterior superior segmental branch of the right renal artery.

Ultrasonography of the right groin identified a patent right femoral artery. Under all sterile precautions, the right common femoral artery was accessed in a retrograde fashion using a 21-gauge micro puncture needle. Using the standard needle, guidewire, and catheter exchange technique, a five-French vascular sheath was placed. The right renal artery was selected using a five-French Cobra catheter. Right renal arteriogram was performed. This showed no extravasation of contrast from the stem of the renal artery and its branches. However, an 8 mm subcapsular aneurysm was identified in the interpolar region. Supersegmental angiography of the posterior superior segmental artery showed the aneurysm likely arising from the interlobular artery. The aneurysm had a filling defect reflecting a thrombus which could be indicative of recent bleeding. The five-French vascular sheath and Cobra catheter were exchanged over a 0.035-inch tiny J-tipped Rosen guidewire for a five-French Balkin sheath. The tip was positioned in the posterior superior segmental artery. Coil embolization of the two interlobar arteries arising from the posterior superior segmental artery was performed. Postembolization angiography revealed contribution to the aneurysm from branches arising from the posterior superior segmental artery. Therefore, additional embolization of the posterior superior segmental artery was performed. Repeat angiography showed no significant flow to the aneurysm, and hemostasis was achieved (Figures 3 and 4).

## 4. Outcome and Follow-Up

The patient had an uneventful recovery following the endovascular intervention. He was later discharged and routinely evaluated three times a week during hemodialysis. He did not develop pain, worsening of anaemia, and hypertension secondary to the iatrogenic renal infarction related to coil embolization of renal artery branches.

## 5. Discussion

### 5.1. Introduction

Renal artery aneurysm (RAA) is a rare entity, with a reported incidence of 0.1% in the general population [1, 2]. Angiographic and computed tomography studies report incidence ranging from 0.3 to 2.5% acknowledging that RAA is being increasingly detected with widespread availability and use of abdominal imaging [2].

### 5.2. Etiology and Natural History

RAA has been associated with hereditary connective tissue dysfunction like fibromuscular dysplasia, Ehler–Danlos syndrome, Marfan syndrome [3], and vasculitis (eg., polyarteritis nodosa and chronic granulomatosis with polyangiitis [4]). Atherosclerosis is also associated with RAA, but the causative role is not defined [5]. RAAs are usually incidentally detected but can present with life-threatening bleeding from aneurysm rupture as seen in our patient. Hypertension is the most frequently associated comorbidity with a reported prevalence as high as 70% [6] and is thought to occur due to coexistent renal artery vaso-occlusive disease, distal embolization, compression, and kinking of renal artery branches especially in the setting of large aneurysm. Our patient had long standing hypertension leading to chronic kidney disease, thus RAA was likely a result from hypertension and atherosclerosis rather than a cause of hypertension. Furthermore, RAA was distally located and smaller in size unlikely to cause renovascular hypertension. There is limited understanding on the risk of RAA rupture. Data are mostly derived from studies in pregnant patients. Risk of RAA rupture is highest in the third trimester of pregnancy and relatively low in postmenopausal female and male patients [7, 8].

Literature on longitudinal surveillance of RAA is far too limited. Our literature search in PubMed, EMBASE, and Scopus revealed three longitudinal studies. A study by Hubert et al. [9] included 62 patients with a mean aneurysm size of 1.5 cm (0.3–4 cm) and followed over a mean duration of 5.7 years. There was no reported event of aneurysm rupture or need for surgery. This study had excluded patients with fibromuscular dysplasia [9]. In a relatively small study by Hernickson et al. [10] in which 21 patients were followed-up for a mean duration of 35 months, there was no reported event of aneurysm rupture, and 82% of RAAs remained anatomically unchanged [10]. However, it is important to note that both of these studies are old and lack survillance data using contemporary imaging techniques. The third and a more recent study by Morita et al. [12] is described later in the discussion.

### 5.3. Indications for RAA Repair

Indications for RAA repair are debatable in absence of the societal guidelines. Studies have advocated the following indications [6–9, 13, 14]:

1. Aneurysm rupture
2. RAA 1–1.5 cm and any of the following:
  - hypertension,
  - female gender (particularly in childbearing age),
  - distal embolization or deterioration of kidney function, and
  - localized flank pain or hematuria thought to be caused by the aneurysm
3. RAA > 2 cm

Aneurysm rupture presents with back pain, abdominal pain, ileus, and hemorrhagic shock as the bleeding progresses. Clinicians should have high index of suspicion as delay in treatment may result in increased morbidity and may lead to nephrectomy, and RAA rupture has a reported mortality as high as 10% [15]. Although surgery was the primary modality in yesteryears, endovascular intervention may be a safer treatment and includes two main modalities, transcatheter embolization or exclusion of the aneurysm using stent grafts.

### 5.4. Literature Review of Treatment Modalitites

We performed literature search in PubMed, EMBASE, and Scopus using the keywords renal artery aneurysm and endovascular intervention. We identified 10 studies published from 2008 to 2016 with a sample size ranging from 3 to 1082. Isolated case reports were excluded. We have summarized the studies in Table 1.

Paschalis-Purtak et al. [15] have reported a case series with 5 patients. Patients were followed-up for a mean duration of 11 months. Complete exclusion of the aneurysm and satisfactory renal artery patency was confirmed in postprocedural follow-up using Doppler sonography and spiral CT.

A retrospective study by Ikeda et al. [16] reports endovascular treatment of 24 visceral artery aneurysms which included 7 patients (mean age 65, 3 males and 4 females) with RAA. 5 RAA were proximal in location and 2 were located distally. 4 patients had coexisting hypertension, and interestingly 3 had rheumatoid arthritis. Although RAA has a reported association with vasculitis like Churg Strauss syndrome and chronic granulomatosis with polyangiitis [4], rheumatoid arthritis is not previously reported, and a definitive causative role of vasculitis has not been established. In this study [16], intervention was performed for the presence of all three factors from 1 to 3 or presence of factor 4 along with at least two factors from 1 to 3 as listed below:

1. Aneurysm more than 1.5 cm in size,
2. No calcification in aneurysm wall
3. No intracavitary thrombus
4. Presence of hypertension, steroid treatment, or pregnancy

None of the interventions were performed in the setting of acute RAA rupture and bleeding. 2 out of 7 patients developed renal infarction due to proximal migration of the coils into the native renal artery. This was detected after the patients developed fever and acute flank pain. Renal infarcts involved less than one third of the renal parenchyma; change in renal function in acute-phase and follow-up period was not reported.

Mannien et al. [13] performed stent-assisted coil embolization in 4 patients (three women, one man; mean age, 54 years; range, 49–67 years) with wide-necked renal artery aneurysms. The stent was delivered over the neck of the aneurysm, after which the aneurysm was filled with detachable coils through a microcatheter placed into the aneurysm through the stent mesh. Stent delivery and coil embolization were successfully completed in all cases. Complete aneurysm occlusion without coil protrusion or arterial flow compromise was obtained in all patients. A small peripheral subsegmental renal infarction necessitating no therapy was registered in one patient.

Hislop et al. [17] identified 215 patients who underwent RAA repair in the New York state from 2001 to 2006 using Statewide Planning and Research Cooperative System database. 91 endovascular and 124 open surgical repairs were performed. Diabetes (15.4% vs 5.6%, *P*=0.018), chronic anaemia (5.5% vs 0.8%, *P*=0.04), and emergent admission (48.4% vs 24.2%, *P* < 0.001) were more prevalent among those patients who were treated with endovascular repair. Endovascular therapy was associated with a lower median length of stay (4 vs 7 days, *P* < 0.001) and lower rates of discharge to skilled nursing facilities (18.9% vs 39.2%, *P*=0.001). Outcomes after endovascular repair were better when compared to conventional surgery, although whether this was due to the technique of repair or preprocedural selection bias could not be determined.

Morita et al. [12], followed 58 patients (17 males and 41 females) with RAA from 1989 to 2009. Median age at presentation was 62 years, and patients were followed-up for a mean duration of 69 months. All 58 patients were screened for extrarenal aneurysms using CT scans and neurosurgical consultation. Extrarenal aneurysms were detected in abdominal aorta (*n*=2), common iliac artery (*n*−2), splenic artery (*n*−1), carotid artery (*n*=1), and cerebral artery (*n*=1). Associated comorbidities included Marfan syndrome (*n*=1), neurofibromatosis (*n*=1), syphillis (*n*=1), and living kidney donor (*n*=1). Patients were divided into two groups, namely, conservative group (*n*=30) who were followed-up with blood pressure control and treatment group (*n*=29) who received either surgical (n−27) or endovascular (*n*=2) intervention. Mean maximum diameter of the RAAs was lower in the conservative group as compared to the treatment group (15 vs 25 mm, *P*=0.05). Two patients in the conservative group showed an increase in the aneurysm size; however, there was no difference in blood pressure control in either group during the follow-up. This study highlights the importance of screening for extrarenal aneurysm as extrarenal aneurysm was seen in 7 (12%) patients.

Abdel-Kerim et al. [18] identified 18 patients with RAA from January 2000 to June 2011. 10 patients were referred to surgery due to a difficult anatomy, and the remaining 8 received endovascular intervention which included selective coil embolization in 5 cases, covered stents in 2 cases, and parent artery occlusion in 1 case. Among the 8 patients, 4 patients were asymptomatic, 3 were hypertensive, and 1 had presented with ipsilateral flank pain. Aneurysmal sac diameter varied from 12 mm to 50 mm. Patients were evaluated with CT angiography during a mean follow-up duration of 15 months (range 6–54 months). Complete occlusion of aneurysm was seen in all patients except 1 who developed reexpansion of the aneurysm 20 months after the procedure. 4 patients developed renal infarction with <25% ischemic renal parenchymal loss. Although patients reportedly had normal renal function at discharge, change in renal function with ischemic renal parenchymal loss during follow-up was not reported.

Zhang et al. [19] reported a seven-year experience (2004–2011) in 15 patients (9 RAA and 6 renal arteriovenous fistulae). Procedures in RAA patients included coil embolization in 7, stent graft in 1, and both in 1 patient. Although no immediate postprocedural complication was seen, postembolization syndrome described as a combination of fever, leukocytosis, abdominal pain, nausea, and vomiting was seen in 3, and partial renal infarction with no loss of renal function seen in 2 patients during a mean clinical follow-up was 24.7 months, and mean imaging follow-up was 16.3 months.

Tsilimparis et al. [20] reported a single center experience of treating renal artery aneurysm from 2000 to 2012. 44 RAA repairs were identified in 40 patients (28 female and 12 male, mean age ± SD, 54 ± 13 years). 20 RAAs were repaired with open surgery (45%) and 24 RAAs (55%) were treated with endovascular repair. Mean aneurysm sizes were 2.5 ± 1.5 cm in the open surgery group vs 2.2 ± 2.2 cm in the endovascular repair group (*P*=0.66). Endovascular repair included coil embolization with or without stent placement in 19 patients (79%) and stent grafts in 4 (17%). Open surgery included excision or aneurysmorrhaphy of the aneurysm in 11 patients (55%), graft interposition or bypass in 4 (20%), and 4 nephrectomies (20%). There was 1 technical failure in each group. Clinical characteristics and comorbidities were similar in both groups.

ASA-PS (American Society of Anesthesiologists Physical Status) Class III and IV were present in 40% of patients in open surgery group compared to 58% in the endovascular repair group (*P*=0.44). No mortality occurred in the either groups. Endovascular repair was associated with shorter hospitalization (open surgery, 6.3 ± 2.5; endovascular repair, 2 ± 3.4 days, *P* < 0.001). Patients were followed-up for 21 ± 32 months in the open surgery group and 27 ± 36 months in the endovascular repair group. This study made a unique observation of 30% reduction in glomerular filtration rate that occurred in 12.5% in the open surgery patients and 9.1% in the endovascular repair group (*P*=1.00).

Li et al. [21] reported 6 cases of ruptured RAA from January 2001 to May 2011. 5 cases were treated with coil embolization and 1 patient was treated with stent graft. Patients were followed-up for a mean duration of 25 months (range 12–64 months); 4 cases with selective coil embolization had complete durable occlusion. Patient treated with trunk artery occlusion developed ischemic parenchymal loss and eventually developed ipsilateral renal atrophy. This study is unique as it has presented all 6 patients with acute RAA rupture.

Buck et al. [22] have published the largest retrospective study on RAAs by reviewing the Nationwide Inpatient Sample from 1988 to 2011. Patients with a primary diagnosis of renal artery aneurysms undergoing open surgery (reconstruction or nephrectomy) or endovascular repair (coil or stent) were identified. Patients with concomitant aortic aneurysms or dissections were excluded. After this exclusion, 6234 RAA repairs were identified in the database from 1988 to 2011 after exclusion; only 2709 patients from 2000 to 2011 were used to compare baseline characteristics and clinical outcome between the open surgery and endovascular repair groups. There were 1627 patients in the open surgery group, and 1082 patients in the endovascular repair group. Patients undergoing endovascular repair were more likely to have a history of coronary artery disease (18% vs 11%; *P* < 0.001), prior myocardial infarction (5.2% vs 1.8%; *P* < 0.001), and renal failure (7.7% vs 3.3%; *P* < 0.001). This suggests that endovascular repair was chosen in face of multiple comorbidities considering the risks of surgery and anesthesia. In-hospital mortality was 1.8% for the endovascular repair group as compared to 0.9% for the open surgery group (*P*=0.037) and 5.4% for nephrectomy (*P* < 0.001 compared with all revascularization). The higher mortality in the endovascular group is likely attributable to the presence of multiple comorbidities. Complication rates were 12.4% for the open repair group vs 10.5% for the endovascular repair group (*P*=0.134), including more cardiac (2.2% vs 0.6%; *P*=0.001) and peripheral vascular complications (0.6% vs 0.0%; *P*=0.014) with open repair. Open repair also had a longer length of stay (6.0 vs 4.6 days; *P* < 0.001). After adjustment for other predictors of mortality, including age (odds ratio (OR), 1.05 per decade; 95% confidence interval (CI), 1.0–1.1; *P*=0.001), heart failure (OR, 7.0; 95% CI, 3.1–16.0; *P* < 0.001), and dysrhythmia (OR, 5.9; 95% CI, 2.0–16.8; *P*=0.005), endovascular repair was still not protective (OR, 1.6; 95% CI, 0.8–3.2; *P*=0.145).

Reviewing the evidence collectively, Morita et al. [12] observed concomitant presence of extrarenal aneurysm in 7 out of 18 (39%) patients. RAA has an etiologic association with hereditary collagen and elastin disorder, vasculitis syndromes, and atherosclerosis. Thus, RAA may be a component of multisystem vasculopathy. Furthermore, in the absence of adequate evidence and lack of societal guideline, this finding may prompt clinicians to evaluate for extrarenal aneurysm based on the clinical context. Clinical and biological behaviour or RAA is not well understood; thus, evidence is deficient to guide clinical follow-up and radiological surveillance. All the listed studies are retrospective and are further limited by short-term follow-up data. Patients were noted to develop postprocedural renal infarcts. These infarcts were seen to produce a decline in renal function in terms of 30% reduction in glomerular filtration rate that occurred in 12.5% in open surgery patients and 9.1% of endovascular repair group (*P*=1.00) in the study by Tsilimparis et al. [20]. Postprocedural renal infarcts were also seen in other studies [16, 18, 19]; however, clinical impact of renal infarcts in terms of decline in glomerular filtration rate and other renal functions parameters was not observed probably due to shorter follow-up period. A significantly lower rate of postoperative complications and a shorter length of stay were seen in endovascular repair [17, 20, 22], but endovascular repair did not show a clear reduction in in-hospital mortality especially considering the largest retrospective study by Buck et al. [22]. Given the operative risk with both endovascular repair and open surgery, reevaluation of the indications for elective repair of isolated renal artery aneurysms is warranted.

### 5.5. Inference

Cardiac risk stratification by revised cardiac risk index (RCRI) in our patient suggested 11% risk for major adverse cardiac event during the perioperative period. Thus, endovascular intervention was chosen given its minimally invasive nature. It is important to note that although endovascular treatment offers a lower postoperative complication and a shorter of length of postprocedural hospitalization, data do not show a clear reduction in mortality [22]. Furthermore, evidence is limited to nonrandomized small retrospective studies; thus, it is hard to draw a conclusion to favor one over the other. Indications of RAA repair and selection of treatment modality needs to be addressed.

## 6. Learning Points/Take Home Messages

1. Clinicians should be well aware of renal artery aneurysm rupture as a potential cause of nontraumatic retroperitoneal hemorrhage.
2. A high index of clinical suspicion is required as delay in diagnosis and intervention can result in a higher morbidity and reported mortality as high as 10%.
3. Endovascular intervention is a minimally invasive modality to treat renal artery aneurysm even in setting of aneurysmal rupture and bleeding. However, evidence is merely limited in small retrospective studies, and large longitudinal prospective studies are required to draw conclusions on long-term outcome.

## Figures and Tables

**Table 1:** Summary of the studies.

Author (Year)	Sample size (*n*)	Endovascular modality	Comorbidity	Outcome
Paschalis-Purtak et al. [15]	*n*=5	3 coil embolization	4 hypertension	No complications
		1 stenting of renal artery		
		1 coil embolization of arteriovenous fistula		
Ikeda et al. [16]	*n*=7	Coil embolization in all 7 patients with additional stent graft in 1 patient	4 Hypertension 3 rheumatoid arthritis	2/7, 29% patients developed renal infarcts due to migration of the coils into native renal artery
Mannien et al. [13]	*n*=4	Stent-assisted coil embolization	NA	A small peripheral subsegmental renal infarction necessitating no therapy was registered in one patient
Hislop et al. [17]	*n*=91	NA		1.1% in-hospital mortality and periprocedural bleeding in 8.8%, renal complications in 2.2%
Morita et al. [12]	*n*=2	Coil embolization in both cases	Marfan syndrome (*n*=1), neurofibromatosis (*n*=1), syphillis (*n*=1), living kidney donor (*n*=1)	No complications
Abdel-Kerim et al. [18]	*n*=8	5 coil embolization	3 hypertension	Aneurysm reexpansion in 1/8 patient, treated with covered stent Renal infarction with <25% parenchymal loss in 4/8 patients
		2 stent grafts		
		1 trunk artery occlusion		
Zhang et al. [19]	*n*=9	7 coil embolization	NA	3/9 (33%) postembolization syndrome 2/9 (22%), partial renal infarct with no loss of renal function
		1 stent graft		
		1 both		
Tsilimparis et al. [20]	*n*=24	19 coil embolization 4 stent grafts	NA	Renal impairment (30% reduction in glomerular filtration rate) reported in 9.1%
Li et al. [21]	*n*=6	5 coil embolization 1 trunk artery occlusion	NA	Ischemic parenchymal loss in trunk artery occlusion
Buck et al. [22]	*n*=1021	NA	NA	1.8% in-hospital mortality 10.5% total complication rate including cardiac 2.2% and peripheral vascular 0.6%
